# Just a Spoonful of Sugar Helps the HSCs Move ’Round

**DOI:** 10.1097/HS9.0000000000000551

**Published:** 2021-03-16

**Authors:** David G. Kent

**Affiliations:** York Biomedical Research Institute, Department of Biology, University of York, York, United Kingdom.

Recent advances in genomic, transcriptomic, and proteomic technologies have permitted fairly comprehensive mapping of the molecules involved in normal and malignant blood stem and progenitor cells. The vast majority of such studies, however, represent a static snapshot of what molecules can be present at a particular time and do not account for molecule turnover, physical positioning, dynamics, or cell:cell interactions among numerous other things. The recent adoption of spatial transcriptomics and larger-scale tissue imaging modalities have permitted the first glimpse into the neighborhoods of individual cells and their molecular features, but the high resolution study of single molecules interacting at tiny scales has been much more challenging.^1,2^ This makes new approaches like the one recently described by Termini et al^3^ so exciting. Exploring the role of proteoglycans and their functional impact on hematopoietic stem and progenitor cells (HSPCs), the authors used stimulated emission depletion (STED) super-resolution imaging to study heparan sulfate proteoglycans and their ability to organize and activate proteoglycan-binding receptors.

Typically, proteoglycans are considered to be critical components of the extracellular matrix with mechanical roles in tissue structure and organizational roles within cellular microenvironments. They regularly exert their function through cell:cell interactions, and this is why it is perhaps a little surprising to be considering them in the context of hematopoietic stem cells. In 2015, the Chute group identified a proteoglycan-binding receptor (PTPσ) which negatively regulated hematopoietic stem cell (HSC) self-renewal,^4^ suggesting that proteoglycans might well have a role to play in influencing the cellular fate of HSPCs. Termini et al^3^ takes a much closer look at this interaction between PTPσ and heparan sulfate proteoglycans using STED super-resolution microscopy, allows researchers for the first time to visualize the nanoscale organization of PTPs on murine bone marrow HSPCs. In this study, the Chute group discovers that Syndecan-2 promotes PTPσ clustering leading to its inactivation. This, in turn, led to ezrin activation and a direct impact on HSPC colony forming ability. While future work will need to show the impact of Syndecan-2 on the long-term self-renewal of HSCs, this still represents an important proof-of-principle experiment demonstrating that proteoglycans found in the extracellular matrix can directly alter HSPC fate without altering the actual amount of PTPσ and simply changing its localization and clustering in the membrane.

These findings make a compelling case for new tools that can study the localization of molecules in the context of cell:cell interactions between HSCs and their in vivo neighbors. Recent efforts to develop HSC reporters should help to identify the key cell types that need to be studied and creates a need for new tools to monitor real time interaction of cells with these neighbors at the single-molecule level to study phenomena such as asymmetric partitioning of molecules upon specific cellular or molecular interactions and the subsequent functional impact of such protein reorganization within a single cell.

## Not just some cool images

Aside from ogling the neat super-resolution images, there appears to also be some clinical value in the assessment of proteoglycans between patients. A recent study published in *Cell Death and Disease* demonstrated that patients with high expression of Heparan sulfate proteoglycan 2, or Perlecan, were much more likely to have worse overall survival and leukemia-free survival.^5^ This implicates it as a potential biomarker for acute myeloid leukemia patients and creates a need to explore the potential role of other extracellular matrix molecules should this prove to be an area of important cellular signaling in the context of malignant hematopoiesis. Hopefully, this article stimulates interest in looking at the cells of the microenvironment for potential biomarkers of a supportive leukemogenic neighborhood rather than keeping all of the focus on the HSPCs themselves.

Together, these 2 papers implicate proteoglycans in both the physical and mechanical properties of a cell and the downstream consequences of specific microenvironmental interactions, as well as explore the potential for such cells and their potentially unique features in the setting of malignant disease. I look forward to seeing additional tools developed in the area of single-molecule interaction with meaningful links through to functional properties of the malignant cells and hope that we continue to move beyond genomic and transcriptomic approaches to molecular medicine.

## Disclosures

The author has no conflicts of interest to declare.
